# Choriocarcinoma Syndrome as an Initial Presentation of Testicular Cancer

**DOI:** 10.1155/2018/8065615

**Published:** 2018-11-08

**Authors:** Carlos Eduardo Salazar-Mejía, María Elena García-Gutiérrez, María Inés Contreras-Salcido, Carlos Javier Rodríguez-Álvarez, Blanca Otilia Wimer-Castillo, Jackeline Grace Lara-Campos, Edio Llerena-Hernández, José Luis González-Vela, David Hernández-Barajas

**Affiliations:** Centro Universitario Contra el Cáncer, University Hospital “Dr. José Eleuterio González” and Faculty of Medicine, Universidad Autónoma de Nuevo León, Monterrey, Nuevo León, Mexico

## Abstract

Choriocarcinoma syndrome (CS) is a rare clinical entity within the spectrum of nonseminomatous germ-cell tumors (NSGCT). It is characterized by the abrupt establishment of rapidly progressive and hemorrhagic tumors associated with very high levels of the beta fraction of human chorionic gonadotropin (*β*-hCG) and with a very poor prognosis, particularly in patients with *β*-hCG values above 50,000 IU/L. We present the case of a 17-year-old man with a sudden onset nonmassive hemoptysis. Physical examination revealed a right testicular mass. Imaging studies showed metastatic lung, bone, and retroperitoneal disease. *β*-hCG serum levels were 222,493.21 IU/L, AFP 1.56 ng/mL, and DHL 457 IU/L. Histopathological study after right radical orchiectomy showed a mixed germ-cell tumor. Based on poor-risk characteristics, chemotherapy was started with an adequate clinical response. Physicians should be aware of the potential complications of CS in the treatment of testicular cancer with high *β*-hCG levels since they could be associated with a rapidly progressive and high-volume disease. Patients in this category should be referred to the centers experienced in the treatment of advanced germ-cell tumors. Due to the severity of the presentation, hemodynamic monitoring, ideally in an intensive care unit, is essential as well as timely administration of cytotoxic treatment.

## 1. Introduction

Choriocarcinoma syndrome (CS) is a clinical entity within the spectrum of advanced nonseminomatous germ-cell tumors (NSGCT) that presents in nearly 10% of these cases. It is characterized by the abrupt establishment of rapidly progressive and hemorrhagic tumors, associated with very high levels of the beta fraction of human chorionic gonadotropin (*β*-hCG) and with a very poor prognosis, particularly in patients with *β*-hCG values above 50,000 IU/L. We describe a rare case of a mixed germ-cell tumor with CS as an initial manifestation.

## 2. Case Presentation

**Figure 1:**
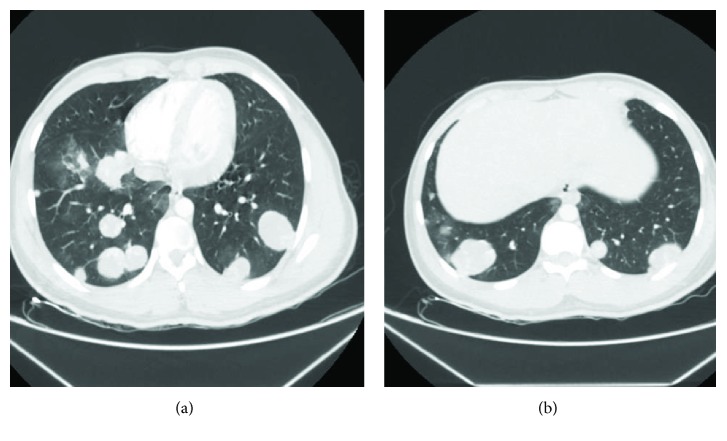


A 17-year-old man was presented to the emergency department with a sudden nonmassive hemoptysis. He had no relevant prior medical history and did not consume alcohol, tobacco, or drugs. On arrival at the emergency room, he was hemodynamically stable, afebrile, and neurologically intact with no need for supplemental oxygen. On physical examination, a stony nodular mass of approximately 0.5 cm on the upper pole of the right testicle which is not painful on palpation was observed. No adenopathies were found. Imaging studies showed multiple round multilobed heterogeneous hypodense lung lesions with a stained glass appearance and poor central enhancement after administration of contrast medium. These were bilateral and randomly distributed; some were subpleural with the appearance of “cannonballs” (Figure 1(a)). The most representative was seen at the level of the upper segment of the right lower lobe, measuring 5.9 × 5.7 × 5.6 cm (Figure 1(b)). At the level of the vertebral body L4, a large adenopathy 4.7 × 3.1 cm with a hypodense center indicative of necrosis was found. This adenopathy compressed the inferior vena cava without compromising its lumen. The right testicle had a heterogeneous appearance with calcifications inside. Serum levels of *β*-hCG were 222,493.21 IU/L, AFP 1.56 ng/mL, and DHL 457 IU/L. A brain MRI showed no relevant alterations. The patient underwent radical right orchidectomy with no complications. A biopsy revealed a mixed multifocal germ-cell tumor 0.3 × 0.2 cm, limited to the right testicle with an embryonic component of 90%, a mature teratoma component of 5%, and a seminoma component of 5%. There was no involvement of the spermatic cord or lymphovascular invasion. After assessment by a multidisciplinary oncology team, it was decided to start a chemotherapy (CT) regimen based on bleomycin, etoposide, and cisplatin for four cycles due to poor-risk characteristics. The patient received his first cycle in a hospital with an adequate clinical response and a favorable tumor marker decline rate. After surveillance, he was discharged to continue ambulatory treatment. At 5 months of follow-up, the patient is alive, receiving second-line CT due to persistent pulmonary disease.

## 3. Discussion

CS was first described in 1984 by Logothetis [1] as a hemorrhagic syndrome produced by NSGCT in advanced stages. It usually occurs in patients with choriocarcinoma and high-volume disease, which is associated with very high *β*-hCG levels. The pathogenesis of this syndrome is unknown; however, it has been postulated that it can be caused by direct tumor invasion of small vessels [2].

The diagnosis of CS is based on clinical suspicion. Acute pulmonary hemorrhage is associated with lung metastases, and respiratory compromise is a typical presentation; however, hemorrhage can occur at any site of metastasis. Liver, small intestine, and CNS involvement are also common in this group of patients [3].

CS has two settings, either hours after the start of combined CT based on bleomycin, etoposide, and cisplatin (BEP) or much less frequently as an initial presentation of advanced disease with no relation to treatment, as in our patient [4]. Symptoms will depend on the site of bleeding; these can be a sudden onset dyspnea, hemoptysis, chest pain, and/or abdominal pain. When the lungs are involved, the symptoms may be similar to diffuse alveolar hemorrhage [4]. Cases of CS as an initial presentation of NSGCT in patients younger than 30 years reported in the English literature are summarized in Table 1. To the best of our knowledge, this is the youngest patient with CS as an initial manifestation of testicular cancer.

Elevated *β*-hCG levels give these patients a poor prognosis according to the International Collaborative Group of Germ Cell Cancer (IGCCCG) published in 1997, which identifies poor-risk disease in patients diagnosed with NSGCT with serum levels of *β*-hCG greater than 50,000 IU/L, showing a progression-free survival and overall survival at 5 years of 41 and 48%, respectively [5]. Due to the low incidence of this presentation, patients with CS are often not identified as a separate group in clinical reports. Currently, there is no specific information on the long-term prognosis of these patients.

In the McKendrick et al. series [6], respiratory symptoms dominated in patients with NSGCT and *β*-hCG levels > 25,000 IU/L. Five patients in this series had hemoptysis at presentation. It is important to mention that choriocarcinoma was demonstrated in the biopsy in only 50% of the patients with these *β*-hCG levels; therefore, CS can exist in patients without a recognized trophoblastic tumor.

The primary treatment of choice for advanced disease consists of three or four cycles of BEP, depending on the risk classification according to the IGCCCG [5]. BEP is the preferred initial regimen for patients with disseminated NSGCT due to its reduced neuromuscular toxicity, a higher percentage of complete responses, and survival in patients with advanced disease [7, 8]. In the case of high risk of development of pulmonary hemorrhage, the VIP regimen (etoposide, mesna, ifosfamide, and cisplatin) could be chosen as first-line CT. In life-threatening hemorrhage, although rare, surgery may be required to avoid a fatal outcome.

Fizazi et al. [10], in a prospective study, evaluated the prognostic relevance of the rate of tumor marker decline during the first three weeks after initiating CT in patients with NSGCT. Tumor markers were taken before and 3 weeks after the first cycle of CT, and it was found that the decline rate, using a logarithmic transformation of the tumoral markers, was a strong predictor of progression-free survival and overall survival in patients with poor-risk NSGCT.

Physicians should be aware of the potential complications that occur with CS during the treatment of testicular cancer with high *β*-hCG levels, which could be associated with a rapidly progressive high-volume disease. Patients in this category should be referred to the centers experienced in the treatment of advanced germ-cell tumors [11]. Due to the severity of the presentation, hemodynamic monitoring, ideally in an intensive care unit, is essential as well as timely administration of cytotoxic treatment.

## Figures and Tables

**Table 1:** Cases of choriocarcinoma syndrome as an initial presentation of NSGCT in patients under 30 years of age reported in the literature.

Author, year, country	Age/origin of hemorrhage	Serum *β*-hCG (IU/L)	Histopathological diagnosis	Outcome/follow-up
Yoshida et al., 2018, Japan [12]	27 years/lung metastasis	943,601	Not specified	Alive/not specified
Salazar-Mejía et al. 2018, Mexico	17 years/lung metastasis	222,493	Mixed germ-cell tumor	Alive, persistent disease/5 months
